# Antioxidant Potential in Different Parts and Callus of *Gynura procumbens* and Different Parts of *Gynura bicolor*

**DOI:** 10.1155/2015/147909

**Published:** 2015-09-30

**Authors:** Vijendren Krishnan, Syahida Ahmad, Maziah Mahmood

**Affiliations:** ^1^Institute of Biosciences, Universiti Putra Malaysia (UPM), 43400 Serdang, Selangor, Malaysia; ^2^Faculty of Biotechnology and Biomolecular Sciences, Universiti Putra Malaysia (UPM), 43400 Serdang, Selangor, Malaysia

## Abstract

Plants from Gynura family was used in this study, namely, *Gynura procumbens* and *Gynura bicolor*. *Gynura procumbens* is well known for its various medicinal properties such as antihyperglycaemic, antihyperlipidaemic, and antiulcerogenic; meanwhile, *G. bicolor* remains unexploited. Several nonenzymatic antioxidants methods were utilized to study the antioxidant capacity, which include ferric reducing antioxidant power (FRAP), 2,2-diphenyl-1-picrylhydrazyl (DPPH) radical scavenging assay, total flavonoid content, total phenolic content, and ascorbic acid content determination. DPPH assay reveals *G. procumbens* shoot as the lowest (66.885%) and *G. procumbens* root as the highest (93.499%) DPPH radical inhibitor. In FRAP assay, reducing power was not detected in *G. procumbens* leaf callus (0.000 TEAC mg/g FW) whereby *G. procumbens* root exhibits the highest (1.103 TEAC mg/g FW) ferric reducing power. Total phenolic content and total flavonoid content exhibited similar trend for both the intact plants analysed. In all antioxidant assays, *G. procumbens* callus culture exhibits very low antioxidant activity. However, *G. procumbens* root exhibited highest phenolic content, flavonoid content, and ascorbic acid content with 4.957 TEAC mg/g FW, 543.529 QE *µ*g/g FW, and 54.723 *µ*g/g FW, respectively. This study reveals that *G. procumbens* root extract is a good source of natural antioxidant.

## 1. Introduction

Plants contain enormous biologically active compounds that contribute as antioxidants. They are secondary metabolites produced to defend against oxidative damage [1] by free radicals which include reactive oxygen species (ROS), reactive chlorine species (RCS), and reactive nitrogen species (RNS). Free radicals rapidly inactivate enzymes, damage cell organelles, and destroy membrane by inducing degradation of proteins lipids and nucleic acid [2]. Furthermore, there is increased evidence that free radicals are the cause for diseases like cancer, diabetes, cardiovascular diseases, autoimmune disorders, neurodegenerative disorders, and ageing [3]. Plants in high environmental stress contain high activities of enzymatic and nonenzymatic antioxidant. Nonenzymatic antioxidants include anthocyanins, ascorbic acid, *α*-tocopherol, *β*-carotene, catechins, coumarins, flavonoids, lignans, and polyphenolic compounds, whereby enzymatic antioxidants include superoxide dismutase (SOD), catalase (CAT), peroxidase (POX), and ascorbate peroxidase (APX) [4, 5]. In addition, synthetic antioxidants are used in food and cosmetic industries which include butylated hydroxyanisole (BHA), butyl hydroxytoluene (BHT), and tert-butylhydroquinone [6]. However, the toxicity and possible carcinogenicity of the synthetic antioxidants were unknown. Antioxidants function to provide cell protection against free radicals produced in response to environmental stress such as salinity, drought, high light intensity, and mineral nutrient deficiency [7]. In practise, application of antioxidants is growing huge in food and beverage industry, cosmetic industry, and nutrition and supplement industry since it is proven to fight against oxidative damages.

Gynura family has been used in traditional medicine in different countries both as systemic and topical application.* Gynura procumbens* specifically has been used in traditional treatment to treat various diseases such as eruptive fever, rashes, kidney diseases, migraines, constipation, hypertension, diabetes mellitus, and cancer [8].* Gynura procumbens* plant offers a vast ethnobotanical benefits which include antiulcerogenic [9], antihypertensive [10], antihyperglycaemic, and antihyperlipidaemic [11] activities. A group of researchers has indicated the presence of multiple classes of compounds in* G. procumbens* extract such as alkaloids, coumarins, flavonoids, triterpenes, and valepotriates. Thus, there is increasing evidence that* G. procumbens* possesses potential medicinal properties. Besides, Akowuah et al. [12] have reported presence of flavonoid like kaempherol-3-0-glucoside, kaempherol-3-0-rutinoside, and quercetin-3-0-rhamnosyl in* G. procumbens* leaf extract. However,* G. bicolor* was not extensively studied for its medicinal properties. Due to that reason,* G. procumbens* (Merr.) and* G. bicolor* which belong to family Asteraceae were studied in this research. This study has attempted to evaluate the antioxidant potential by mean of five different methods which include FRAP assay, DPPH free radical scavenging assay, total phenolic content, total flavonoid content, and ascorbic acid content on different parts and callus of* G. procumbens* and different parts of* G. bicolor*. The findings of this analysis are crucial in further understanding and development of this plant to be used in treatment of diseases.

## 2. Experimental

### 2.1. Plant Materials

*G. procumbens* and* G. bicolor* plants were collected from University Agriculture Park and maintained in the glass house. Plants of fully expanded leaves without any defect were randomly chosen for the experiments.

### 2.2. Chemicals

2,2-Diphenyl-1-picrylhydrazyl (DPPH), 2,4,6-tripyridyl-s-triazine (TPTZ), Folin Ciocalteu phenol reagent, Trolox, and gallic acid were purchased from Sigma Co. (St. Louis, Missouri, USA). Sodium nitrite, acetic acid, sodium hydroxide, aluminium chloride, gallic acid, iron(III) chloride hexahydrate, and sodium carbonate were purchased from Merck, Darmstadt, Germany. All chemicals and reagents used were of analytical grade.

### 2.3. Sample Preparation

One gram of fresh root, stem, and leaf were crushed mechanically using mortar and pestle individually. The crushed samples were then extracted with 25 mL of double deionised water by shaking the suspension continuously on orbital shaker for an hour. The extracts were filtered through vacuum filtration by using Whatman filter paper number 1. Then, the extract was concentrated to 5 mL with rotary evaporator at 40°C. The extract was then stored in brown glass bottle at −20°C for further use.

### 2.4. DPPH Free Radical Scavenging Assay

Free radical scavenging effect was evaluated using DPPH radical based on method as described by Brand-Williams et al. [13]. DPPH radical (0.8 mM) solution in 95% ethanol was prepared. Plant extract (400 *μ*L) was diluted to 5 mL using distilled water and ethanol (1 : 1) before 1.0 mL DPPH solution was added and shaken vigorously. The absorbance of the mixture was recorded after 10 min at 515 nm against a blank of ethanol without DPPH. A calibration curve was prepared based on Trolox (10, 20, 30, 40, and 50 *μ*g, *R* ^2^ = 0.970). The percentage of DPPH radical inhibition was calculated according to the following equation:

(1) Percentage  inhibition  of  DPPH=Absorbance  control−Absorbance  sampleAbsorbance  control×100.

Absorbance control is the absorbance of DPPH solution without extract.

### 2.5. Ferric Reducing Antioxidant Power (FRAP) Assay

Ferric reducing antioxidant power assay, a method for measuring total reducing power of electron donating substances, was performed according to modified method described by Benzie and Strain [14]. Briefly, 100 *μ*L of plant extract was mixed with 3 mL of FRAP reagent. The FRAP reagent should be prewarmed at 37°C and should always be freshly prepared by mixing 2.5 mL of 10 mM TPTZ solution in 40 mM hydrochloric acid with 2.5 mL of 20 mM FeCl_3_·6H_2_O and 25 mL of 0.3 M sodium acetate buffer pH 3.6. Then, the reaction mixture was incubated at 37°C for 4 min. After that, the absorbance was determined at 593 nm against a blank that was prepared using distilled water and incubated for an hour instead of 4 min. A calibration curve was prepared using an aqueous solution of Trolox (10, 20, 30, 40, and 50 *μ*g, *R* ^2^ = 0.994). FRAP values were expressed on a fresh weight basis as milligram (mg) of Trolox equivalents per gram of sample.

### 2.6. Total Phenolic Content (TPC)

Total phenolic content of the plant extract was determined using Folin Ciocalteau (FC) assay which was described by Singleton and Rossi Jr. [15]. Plant extract (200 *μ*L) was pipetted into test tubes. Then, 1.8 mL of FC reagent was prediluted 10 times with distilled water introduced into respective test tubes. After standing for 5 min at room temperature, 1.2 mL of (7.5% w/v) sodium carbonate solution was added and mixed well. The solution was allowed to stand for an hour at room temperature, before absorbance was measured at 765 nm using UV-visible spectrophotometer (Shimadzu, Japan). A calibration curve was prepared using a standard solution of gallic acid (20, 40, 60, 80, and 100 mg/mL, *R* ^2^ = 0.995). Total phenolic content was expressed on fresh weight basis as milligram gallic acid equivalents per gram of sample.

### 2.7. Total Flavonoid Content (TFC)

Total flavonoid content of the plant extract was performed according to the colorimetric assay developed by Zhishen et al. [16]. Plant extract (500 *μ*L) was diluted with 4.5 mL of distilled water. At zero time, 0.3 mL of (5% w/v) NaNO_2_ was introduced into test tubes containing the diluted extract. After 5 min, 0.3 mL of (10% w/v) AlCl_3_ was added into the solutions. At 6 min, 2 mL of 1.0 M NaOH solution was added to the mixture solution. The solution was then diluted by addition of 2.4 mL distilled water to make the final volume up to 10 mL. The solution was shaken vigorously and the absorbance of the mixture was measured at 570 nm using a UV-visible spectrophotometer (Shimadzu, Japan). A calibration curve was prepared using a standard solution of kaempherol (20, 40, 60, 80, and 100 mg/mL, *R* ^2^ = 0.992). Total flavonoid content was expressed on a fresh weight basis as microgram kaempherol equivalents per gram of sample.

### 2.8. Ascorbic Acid Content

Spectrophotometry method using dichlorophenolindophenol (DCPIP) was performed according to modified method as described by Davies and Masten [17] to determine ascorbic acid content. Plant extract (1.0 mL), 1.0 mL of 1.5 M acetic acid, 1.0 mL of 0.25 M Na_2_H_2_EDTA in 0.375 M NaOH, and 0.2 mL of 0.3 mg/mL DCPIP solution were mixed and diluted to 5.0 mL with distilled water. Then, the absorbance at 520 nm was read within 10 min of mixing the reagents. The DCPIP solution was added immediately after adding the Na_2_H_2_EDTA-NaOH solution to prevent autooxidation of ascorbic acid. A calibration curve was prepared using a standard solution of ascorbic acid (20, 40, 60, 80, and 100 mg/mL, *R* ^2^ = 0.986). Ascorbic acid content was expressed on a fresh weight basis as microgram ascorbic acid per gram of sample.

### 2.9. Statistical Analysis

All experiments were replicated thrice, mean values were pooled, and standard deviations (SD) were calculated. Analysis of variance (ANOVA) and Duncan's Test were carried out on the values obtained in the experiment by using software SPSS V19.0.

## 3. Results

**Figure 1:**
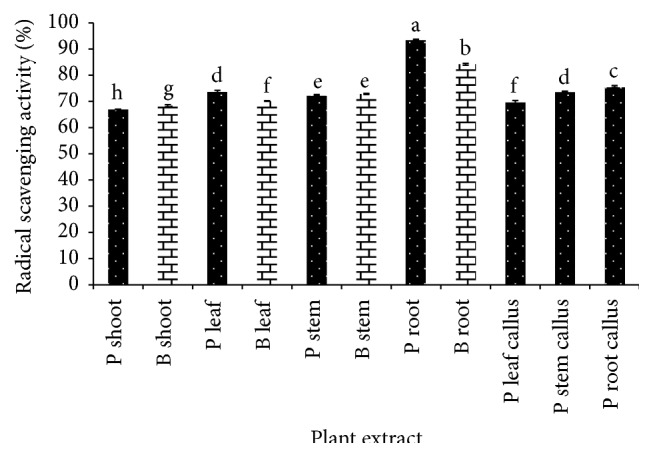
Total antioxidant content of* G. procumbens* (P) and* G. bicolor* (B) plant parts and callus cultures expressed as percentage of inhibition using DPPH assay. Values are means ± S.D. (*n* = 3).

The results of radical scavenging effects determined by DPPH assay are shown in Figure 1. In general, inhibition percentages of DPPH are ranging from 66.89 to 93.50%. In comparison to the various parts and callus of* G. procumbens* and* G. bicolor*, shoot of* G. procumbens* showed the least inhibition percentage indicating less effectiveness in radical scavenging with 66.89% and followed by* G. bicolor* shoot which exhibit 68.33% radical inhibition. Root of* G. bicolor* and* G. procumbens* possesses the highest radical scavenging activity among the extracts with 72.86 and 84.00%, respectively. Thus, the percentage of DPPH inhibition can be sequenced in the following order: P root > B root > P root callus > P stem callus > P leaf > B stem > P stem > B leaf > P leaf callus > B shoot > P shoot.

**Figure 2:**
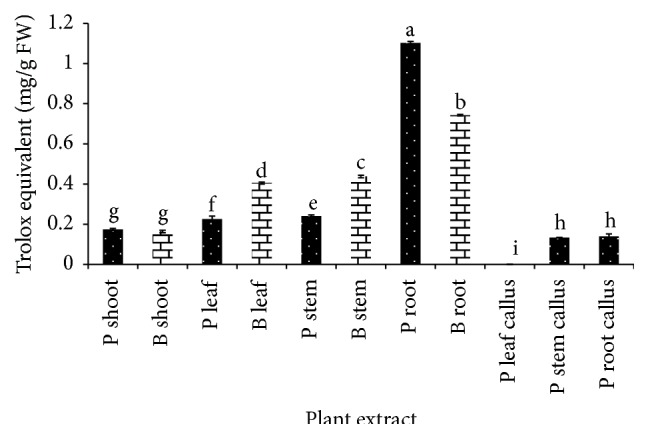
Total antioxidant content of* G. procumbens* (P) and* G. bicolor* (B) plant parts and callus cultures expressed as Trolox equivalent antioxidant capacity (mg/g FW) using FRAP method. Values are means ± S.D. (*n* = 3).

FRAP assay is commonly used to determine antioxidant capacity in fruits, vegetables, and medicinal herbs. Root extract of* G. procumbens* and* G. bicolor* shows outstanding ferric reducing ability compared to other extracts (Figure 2). Both plants show similar trend of ferric reducing ability in the sequence of shoot, leaf, stem, and root. Like plant parts,* G. procumbens* callus exhibits increasing ferric reducing power in sequence of leaf, stem, and root callus. However, among all extracts* G. procumbens* leaf callus, stem callus, and root callus demonstrated lowest reducing ability with 0.000, 0.133, and 0.140 TEAC mg/g FW, respectively. Interestingly, root of* G. procumbens* exhibited strongest antioxidant capacity with 1.103 TEAC mg/g FW followed by root of* G. bicolor* with 0.743 TEAC mg/g FW. Total antioxidant capacity from both DPPH and FRAP assays does not show similar trend; this could be due to different mechanism of assay method, structure of different phenolic compounds, the antioxidant protection mechanism exhibited by compounds, and also the synergistic effects of different compounds [18]. Correlation coefficient analysis was performed between DPPH and FRAP which resulted in relatively high correlation (*r* = 0.886) (Table 1).

**Figure 3:**
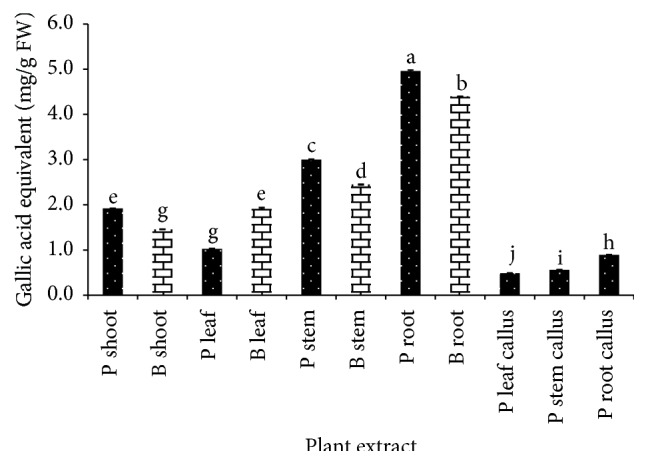
Total phenolic content of* G. procumbens* (P) and* G. bicolor* (B) plant parts and callus cultures expressed as gallic acid equivalent (mg/g FW) in various intact parts. Values are means ± S.D. (*n* = 3).

Folin Ciocalteau's assay is one of the oldest methods developed to determine total phenolic content in vegetables, fruits, and medicinal plants [19]. Phenolic compound in basic condition dissociates into phenolate anion which is capable of reducing FC reagent in which the molybdate is reduced forming a blue coloured molybdenum oxide with maximum absorption near 750 nm [20]. The TPC determined by FC method varied from 0.483 to 4.957 GAE mg/g FW (Figure 3).* Gynura procumbens* leaf callus, stem callus, and root callus extract exhibited the lowest phenolic content with 0.483, 0.559, and 0.891 GAE mg/g FW, respectively. Meanwhile, roots of* G. procumbens* and* G. bicolor* show highest TPC with 4.957 and 4.389 GAE mg/g FW.

**Figure 4:**
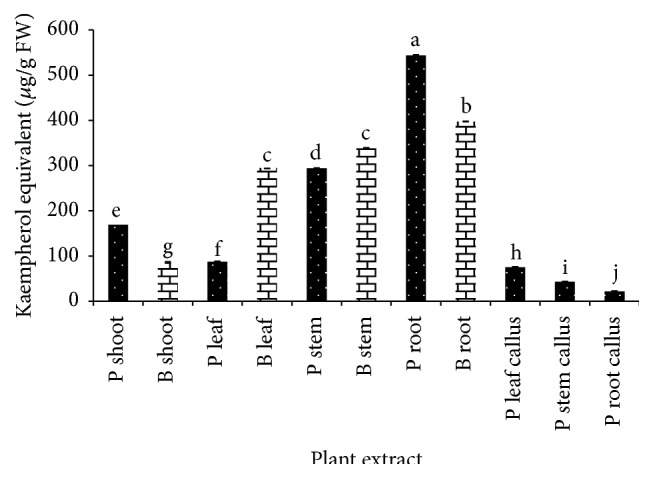
Total flavonoid content of* G. procumbens* (P) and* G. bicolor* (B) plant parts and callus cultures expressed as kaempherol equivalent (*μ*g/g FW). Values are means ± S.D. (*n* = 3).

Total flavonoid content was determined by colorimetric assay method developed by Zhishen et al. [16]. Flavonoid content in both plants varied from 21.961 to 543.529 KE *μ*g/g FW (Figure 4).* Gynura procumbens* leaf callus, stem callus, and root callus extract exhibited the lowest TFC among all extracts with 75.258, 43.351, and 21.961 KE *μ*g/g FW, respectively. Meanwhile,* G. procumbens* root extract shows the highest TFC with 543.529 KE *μ*g/g FW. Interestingly, high correlation coefficient was found between total phenolic content and total flavonoid content (*r* = 0.944).

**Figure 5:**
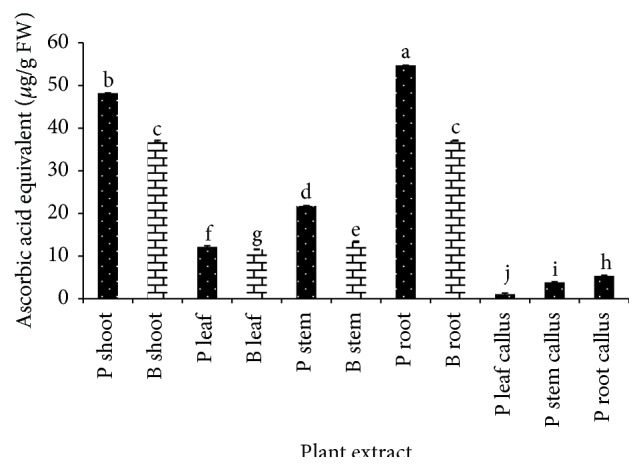
Total ascorbic acid content of* G. procumbens* (P) and* G. bicolor* (B) plant parts and callus cultures expressed as ascorbic acid equivalent (*μ*g/g FW). Values are means ± S.D. (*n* = 3).

Ascorbic acid contents for* G. procumbens* and* G. bicolor* (Figure 5) were determined by DCPIP based on decrease in the absorption of the protonated form of the DCPIP dye at 520 nm due to its reaction with ascorbic acid. Among all the extracts,* G. procumbens* leaf callus, stem callus, and root callus exhibit low ascorbic acid content with 1.069, 3.844, and 5.352 *μ*g/g FW, respectively. Interestingly,* G. procumbens* and* G. bicolor* shoot which exhibited low activity in FRAP, TPC, and TFC, show high ascorbic acid content. In addition,* G. procumbens* root possesses the highest ascorbic acid content with 54.723 *μ*g/g FW, followed by* G. procumbens* shoot with 48.178 *μ*g/g FW. This result shows that root and shoot part of* G. procumbens* and* G. bicolor* possess high ascorbic acid content.

Due to the fact that phenolic compounds, flavonoid compounds, and ascorbic acid contribute to antioxidant capacity, the correlation coefficient analysis was also performed to correlate the total phenolic content, total flavonoid content, and ascorbic acid content with DPPH and FRAP. A strong correlation between FRAP assay and total phenolic content as well as total flavonoid content was observed which implies that antioxidants in both these plants are highly capable of reducing oxidants, whereby correlation coefficients observed between total phenolic content and total flavonoid content with FRAP are *r* = 0.915, *r* = 0.911, respectively. On the other hand, moderate correlations were obtained between DPPH and total phenolic content and total flavonoid content indicating low capability to scavenge free radicals, whereby total phenolic content and total flavonoid content exhibit correlation coefficient with DPPH (*r* = 0.762, *r* = 0.691, resp.). In addition, poor correlation was obtained between AAC and DPPH as well as FRAP, indicating that ascorbic acid does not strongly scavenge free radicals and reduce oxidants. It is also important to note that total phenolic content correlates slightly better than total flavonoid content to DPPH, which shows that phenolic compounds present in the plant are the major contributor to the radical scavenging activity. This result is in agreement with Kumaran and Karunakaran [21] and Lu et al. [22]. Based on the results obtained, all extracts of* G. procumbens* and* G. bicolor* attributed to the presence of antioxidant compound. Investigation carried out has shown that root extract of* G. procumbens* is a more potential medicinal source to treat diseases since it possesses higher antioxidant activity as well as higher phenolic compounds, higher flavonoid contents, and ascorbic acid content. However, this finding contradicted the traditional medicine where leaf was used vastly to treat diseases.

## 4. Discussion

Phytochemical compounds are commonly found in edible and nonedible plants and they have been reported to have multiple biological effects including antioxidant activity. There are various antioxidant compounds present in plants and it is very difficult to measure each antioxidant activity separately. In this research, only water soluble antioxidants have been determined. However,* G. procumbens* and* G. bicolor* have been reported with presence of lipophilic antioxidant compounds such as carotenoids [23, 24]. Carotenoids including *β*-carotene, lutein, and zeaxanthin are part of antioxidant defence system, in which they interact synergistically with other antioxidants to scavenge reactive oxygen species, singlet molecular oxygen, and peroxyl radicals [25]. Böhm et al. [26] have reported that *α*-tocopherol, ascorbic acid, and *β*-carotene exhibited cooperative synergistic effects better than individual antioxidants on scavenging reactive nitrogen species.

Karadeniz et al. [27] in their research have shown that all of the fruits and vegetables contain antioxidant activity although they vary in their activity. Therefore, several methods have been established to measure the antioxidant activity of biological materials. Among them are ferric reducing antioxidant power (FRAP) [14], DPPH radical scavenging assay [13], ABTS radical cation decolorization assay [28], and oxygen radical absorbance capacity assay (ORAC) [29]. Two commonly used methods, FRAP and DPPH which are reliable, rapid, easy, and accurate to determine the ability of antioxidant, were used in this study [30]. FRAP assay is based on the reducing power of the biological materials. Antioxidant capacity of compounds was determined by the reducing potential of an antioxidant reacting with ferric tripyridyltriazine (Fe^3+^-TPTZ) complex and to produce a blue coloured complex of ferrous tripyridyltriazine (Fe^2+^-TPTZ) [31]. Ferrous tripyridyltriazine in FRAP reagent has intense blue colour and can be read at absorbance 593 nm [32].

DPPH assay is based on measurement of antioxidant ability to scavenge the stable DPPH radical. DPPH is a stable nitrogen centred free radical, which produces violet colour in methanol solution. DPPH radicals react with suitable reducing agents during which the electrons become paired off and solution loses colour stoichiometrically depending on number of electrons taken up [3]. Also, rapid decrease in absorbance indicates higher potency of the antioxidant compound.

Antioxidant activities of different parts of plants vary depending on the mechanism and function of the phenolic compounds. In addition, antioxidant activity also varies among plant species and family. As described by Tomaino et al. [33], contents of phenolic compounds were significantly higher in skins than in seeds of* Pistacia vera *L. (pistachio nuts). In addition, scavenging effect varies among plant family and species [34], whereby plants of same family* Platypodium elegans* and* Pseudopiptadenia contorta* exhibited different scavenging effects. Leaves of* P. elegans* possess higher free radical scavenging ability than leaves of* P. contorta*.

In a research conducted on* Centella asiatica*, the leaves showed highest antioxidant activity compared to petiole and roots by mean of ferric thiocyanate (FTC) method and thiobarbituric acid (TBA) assay [13]. This is in accordance with Mensor et al. [34], whereby free radical scavenging effects of leaves* Lantana trifolia* and* Lantana camara* were lower than their barks. However, Siddhuraju et al. [35] have reported higher antioxidant activity of stem bark compared to leaves, flowers, and pulp. Thus, antioxidant activity analyses of various parts of plants and species need to be carried out in order to obtain the accurate measurements. Finding from this study is also in accordance with Jaleel [5] whereby roots of* Withania somnifera* exhibited higher antioxidant activity compared to its leaves by mean of enzymatic and nonenzymatic antioxidant.

## 5. Conclusion

High antioxidant activity is observed in the roots of* G. procumbens* as compared to other extracts tested. Thus, this extract can be considered as a new source of natural antioxidant for disease healing and health supplements.

## Figures and Tables

**Table 1:** Pearson bivariate correlation coefficients (*r*) between different antioxidant assays of intact plant parts and callus of *G. procumbens* and *G. bicolor*.

Correlation coefficient, *r*	DPPH	FRAP	TPC	TFC	AAC
DPPH	1	0.886	0.762	0.691	0.440
FRAP		1	0.915	0.911	0.630
TPC			1	0.944	0.727
TFC				1	0.592
AAC					1

## Abbreviations

AAC: Ascorbic acid content
DPPH: 2,2-Diphenyl-1-picrylhydrazyl
FC: Folin Ciocalteau
FW: Fresh weight
QE: Quercetin equivalent
TEAC: Trolox equivalent antioxidant capacity
TFC: Total flavonoid content
TPC: Total phenolic content
TPTZ: 2,4,6-Tri(2-pyridyl)-s-triazine.
